# Microsimulation Model for Prevention and Intervention of Coloretal Cancer in China (MIMIC-CRC): Development, Calibration, Validation, and Application

**DOI:** 10.3389/fonc.2022.883401

**Published:** 2022-04-22

**Authors:** Bin Lu, Le Wang, Ming Lu, Yuhan Zhang, Jie Cai, Chenyu Luo, Hongda Chen, Min Dai

**Affiliations:** ^1^ Office of Cancer Screening, National Cancer Center/National Clinical Research Center for Cancer/Cancer Hospital, Chinese Academy of Medical Sciences and Peking Union Medical College, Beijing, China; ^2^ Medical Research Center, Peking Union Medical College Hospital, Chinese Academy of Medical Sciences and Peking Union Medical College, Beijing, China; ^3^ Department of Cancer Prevention, Cancer Hospital of the University of Chinese Academy of Sciences (Zhejiang Cancer Hospital), Institute of Cancer and Basic Medicine, Chinese Academy of Sciences, Hangzhou, China; ^4^ Department of General Surgery, Peking Union Medical College Hospital, Chinese Academy of Medical Sciences and Peking Union Medical College, Beijing, China

**Keywords:** microsimulation model, colorectal cancer, natural history, screening, Markov model

## Abstract

**Introduction:**

A microsimulation model provides important references for decision-making regarding colorectal cancer (CRC) prevention strategies, yet such a well-validated model is scarce in China.

**Methods:**

We comprehensively introduce the development of MIcrosimulation Model for the prevention and Intervention of Colorectal Cancer in China (MIMIC-CRC). The MIMIC-CRC was first constructed to simulate the natural history of CRC based on the adenoma-carcinoma pathway. The parameters were calibrated and validated using data from population-based cancer registry data and CRC screening programs. Furthermore, to assess the model’s external validity, we compared the model-derived results to outcome patterns of a sigmoidoscopy screening trial in the UK [UK Flexible Sigmoidoscopy Screening (UKFSS) trial]. Finally, we evaluated the application potential of the MIMIC-CRC model in CRC screening by comparing the 8 different strategies.

**Results:**

We found that most of the model-predicted colorectal lesion prevalence was within the 95% CIs of observed prevalence in a large population-based CRC screening program in China. In addition, model-predicted sex- and age-specific CRC incidence and mortality were equivalent to the registry-based data. The hazard ratios of model-estimated CRC-related incidence and mortality for sigmoidoscopy screening compared to no screening were 0.60 and 0.51, respectively, which were comparable to the reported results of the UKFSS trial. Moreover, we found that all 8 strategies could reduce CRC incidence and mortality compared to no screening.

**Conclusions:**

The well-calibrated and validated MIMIC-CRC model may represent a valid tool to assess the comparative effectiveness of CRC screening strategies and will be useful for further decision-making to CRC prevention.

## Introduction

In China in 2020, newly diagnosed cases and deaths of colorectal cancer (CRC) were estimated to be 555,477 and 286,162, respectively (1), which are expected to continue to rise in the coming decades (2). Population-based screening has been demonstrated to be effective in containing the upward trends of CRC (3–5). While several CRC screening programs have been implemented in some areas since 2005 (6), few studies have evaluated the long-term effectiveness of CRC screening in China (7, 8).

To avoid drawbacks of long duration and high cost for cohort studies and demanding randomized controlled trials (RCTs), several countries have adopted model simulation to evaluate the effect of screening strategies (2, 9–12). Microsimulation models, which simulate individual disease history using stochastic parameters describing transitions between specified health states, are becoming common in the field of decision-making for health policy. Through changing the life histories of a large population of individuals, a microsimulation model can be used to estimate the effects of interventions and policies on the population. The results from Cancer Intervention and Surveillance Modeling NETwork (CISNET) project have assisted policymakers in decision-making relative to CRC screening guidelines (13, 14). Compared with traditional epidemiological evaluation designs, microsimulation models are a valuable tool to provide a relatively inexpensive and flexible way to explore the impact of different interventions and policy changes on CRC incidence and mortality.

Although cohort studies and RCTs could provide high-level evidence in evaluating the long-term effectiveness of screening strategies, the implementation time of nationally representative screening programs is too short to provide long-term evidence in China. In that sense, a microsimulation model study is a satisfying method to address the issue. There are few simulation models for CRC screening in China (15–17), none of which were well-calibrated and well-validated. The goal of the present study is to comprehensively describe the construction, calibration, and validation of a multistate microsimulation model in China and to perform the application of the model by comparing CRC screening effects in different screening scenarios. This study followed the Strengthening the Reporting of Empirical Simulation Studies (STRESS) reporting guideline (18).

## Materials and Methods

**Figure 1:**
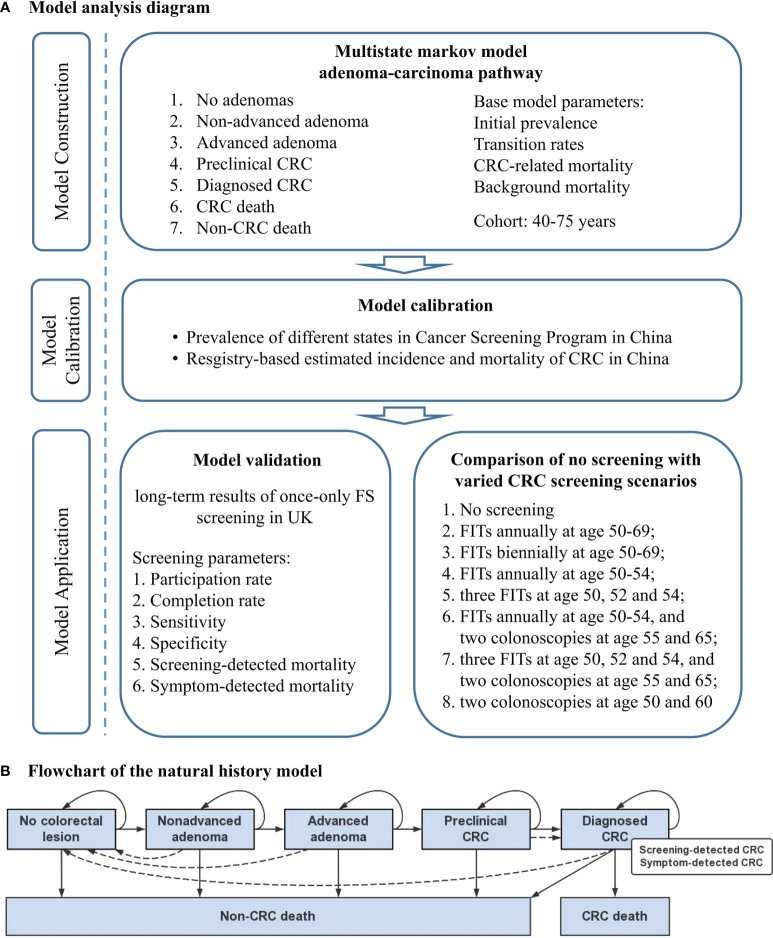
Concept framework and analysis flow. **(A)** Model analysis diagram. **(B)** Flowchart of the natural history model for the adenoma-carcinoma sequence. Solid lines represent the progression of colorectal lesions through the adenoma-carcinoma sequence in the absence of screening; dashed lines show the movement between states because of the detection and removal of adenoma, the detection of asymptomatic colorectal cancer by screening or symptom, and the curation of colorectal cancer. CRC, colorectal cancer; FS, flexible sigmoidoscopy; FIT, fecal immunochemical test.

The analysis diagram of MIcrosimulation Model for prevention and Intervention of Colorectal Cancer in China (MIMIC-CRC) is shown in **Figure 1A** and was conducted between October 1, 2020, and December 31, 2021. The model was constructed using TreeAge pro Healthcare Version 2021 R1.1, and further statistical analyses were performed using R version 6.0.

### Model Construction

MIMIC-CRC simulates the individual health trajectory of a specific birth cohort from 40 to 80 years using a microsimulation approach. Given that 70%–90% of CRC originated from the adenoma-carcinoma pathway and the lack of research source about serrated neoplasia pathway and microsatellite instability pathway in China (19), our model is based solely on the adenoma-carcinoma sequence and assumes that all CRC cases arise from adenoma. Seven distinct states are used to describe the natural history of CRC: 1) no colorectal lesion, 2) non-advanced adenoma (NAA), 3) advanced adenoma (AA), 4) preclinical CRC, 5) diagnosed CRC (categorized into screening-detected CRC and symptom-detected CRC), 6) CRC-related death, and 7) non-CRC death. The detailed model structure is shown in **Figure 1B**. Simulations are performed at discrete time steps of 1 year with individual states changing along the direction of arrows following the assumptions: 1) under natural conditions, the states of individuals can only progress rather than regress; 2) if naturally transit to CRC, preclinical CRC will eventually turn to symptom-detected CRC; 3) if detected by colonoscopy, NAA and AA will transit to no colorectal lesion state, and preclinical CRC will be regarded as screening-detected CRC; 4) CRC-related death only occurs in individuals with diagnosed CRC; 5) individuals diagnosed of CRC who survive for more than 5 years will be regarded as survivors. State transitions within an individual are modeled independently from each other. We hypothesize that an individual may only develop a colorectal lesion at the same time, and the final diagnostic status is defined according to the most severe lesion.

Parameterization is the fundamental part of the model construction. To compare the effectiveness of different screening modalities, we simulate a specific fixed-cohort from the age of 40 years in 2015 (who were born in 1975) until they are dead or have reached the age of 80. In this model, parameters are divided into two parts, including natural history parameters and screening-related parameters. Natural history parameters consist of four main components: initial prevalence rates, transition probabilities between disease states, probabilities of CRC-related death, and probabilities of non-CRC death (see **Supplementary Table 1**). The screening-related parameters include the main diagnostic indicators of respective examinations, which will be described in the subsequent section.

Initial prevalence rates, which are the distributions of the population with CRC-related states at the age of 40 years, were obtained from a large-scale multicenter population-based colonoscopy screening program for CRC in China (20). Apart from a population with CRC or death of which the default initial prevalence is 0, people with no colorectal lesion, NAA, AA, and preclinical CRC have sex-specific initial prevalence. The annual transition probabilities between CRC-related states were estimated based on published studies concerning the natural history of CRC (9). Considering the variations of CRC incidence by gender and age group, we hypothesized that transition probabilities also differed by gender and age. The un-adjusted annual probabilities of CRC-related death come from the study by Heisser et al. in Germany (21) because of the lack of available respective data in China. Given the difference of overall survival rates between China and Germany [5-year relative survival rate 52.7% in 2006–2008 (22) vs. 62.2% in 2000–2007 (23)], the annual probabilities of CRC-related death were all adjusted. The annual sex- and age-specific probabilities of non-CRC death were calculated according to the formula P = 1 − exp[−(P_all-cause_ − P_CRC_)], where P_all-cause_ is sex- and age-specific all-cause mortality and P_CRC_ is sex- and age-specific CRC-related mortality. The all-cause mortality and CRC-related mortality were derived from the China Health & Family Planning Statistics Yearbook 2016 (see **Supplementary Table 2**) (24).

### Model Calibration

Calibration is vital for ensuring the reliability of model parameters. Briefly, the calibration procedure aimed at finding parameter sets that produced intermediate model outcomes fell within the satisfactory interval of the observed data. The parameters obtained by calibration are shown in **Supplementary Table 1**. First, the transition probabilities were calibrated against the age-specific distribution of CRC-related states from the study by a large population-based CRC screening program in China (20). In addition, we calculated the rate ratios (RRs) of model-estimated incidence and mortality divided by the rates observed in China from Global Burden of Disease (GBD) 2019. Statistical equivalence between the modeled and observed rates was tested by applying 2 one-sided t-tests (TOSTs) (25) to each of the sex-specific meta-analysis estimates at a 20% equivalence margin.

### Model Validation

After calibration, we simulated an RCT by modeling populations with and without one-time sigmoidoscopy screening, to assess the magnitude of reduction of CRC incidence and mortality by sigmoidoscopy screening. The objective was to assess the concordance of the model-estimated results compared to the results of the UK Flexible Sigmoidoscopy Screening (UKFSS) trial (26, 27). UKFSS trial aimed to examine the hypothesis that one-off flexible sigmoidoscopy (FS) screen for the population aged 55 to 64 years is a cost-effective and acceptable method to reduce CRC incidence and mortality as compared with no screening. We simulated 112,939 individuals with usual care as the control group and 40,621 individuals with one-off FS between the age of 55 and 64 years as the screened group based on the actual situation in the UKFSS trial according to attendance for screening (per-protocol analysis). Between both groups, we compared the cumulative hazard ratio (HR) of incidence, CRC-related mortality, and other-cause mortality after the end of follow-up. The total duration of the model simulation was set to 11.2 years based on the median follow-up time in the trial. The sensitivities of FS for adenoma and CRC were estimated to be 0.59 and 0.61, respectively, with the specificity of 0.92 according to a Meta-analysis by Niedermaier et al. (see **Supplementary Table 3**) (28). We hypothesized all the NAA cases detected by FS were removed, which was consistent with the criteria in the trial. Referral for colonoscopy was required when any AA or CRC was screened by FS and the completion rate of colonoscopy was 96% (27).

### Model Application

To evaluate the potential application of the model in secondary prevention of CRC, comparisons of the long-term incidence and mortality reduction of different screening strategies versus no screening were modeled. For this analysis, the following 8 different screening scenarios each containing 100,000 individuals were carried out: 1) no screening; 2) fecal immunochemical tests (FITs) annually at age 50–69; 3) FITs biennially at age 50–69; 4) FITs annually at age 50–54; 5) three FITs at age 50, 52, and 54; 6) FITs annually at age 50–54, and two colonoscopies at age 55 and 65; 7) three FITs at age 50, 52, and 54, and two colonoscopies at age 55 and 65; and 8) two colonoscopies at age 50 and 60. In this analysis, individuals were referred to diagnostic colonoscopy if the FIT test was positive and the completion rate of colonoscopy was 0.76. Sensitivity values of FIT for detecting NAA, AA, and CRC were 0.05, 0.26, and 0.76 (29), respectively, with the specificity of 0.95 (30). Among the screening scenarios, HRs of cumulative CRC incidence and mortality were used as comparative indicators. All analyses were conducted using a real-world screening participation rate of 94% for FIT screening and 42.5% for colonoscopy screening, according to a large-scale multicenter RCT in China (31). For individuals with negative findings at colonoscopy screening, a colonoscopy will be offered at a 10-year interval according to the current guidelines (32).

### Sensitivity Analyses

To account for uncertainty related to assumptions on parameters in the model, we conducted sensitivity analyses for the comparison of the efficacy of different screening strategies. One-way sensitivity analyses were implemented by adjusting point estimates of different parameters (see **Supplementary Table 4**).

## Results

### Model Calibration

**Figure 2:**
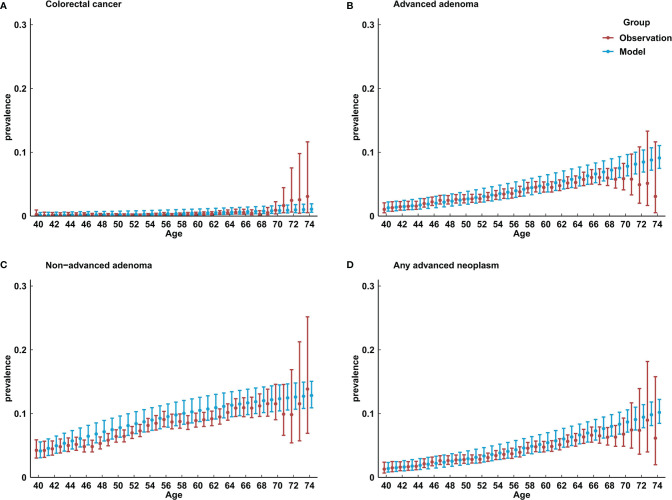
Comparison of model-predicted prevalence of colorectal lesions with the observed results in China. **(A)** prevalence of colorectal cancer; **(B)** prevalence of advanced adenoma; **(C)** prevalence of non-advanced adenoma; **(D)** any advanced neoplasm.

**Figure 2** shows the comparison of model-predicted prevalence of colorectal lesions with the observed prevalence in a large-scale population-based screening colonoscopy program in China in 2012–2015 (20). A large majority (overall, 124 out of 140) of model-predicted CRC, AA, NAA, and any advanced neoplasm prevalence were within the 95% CIs of observed prevalence.

**Figure 3:**
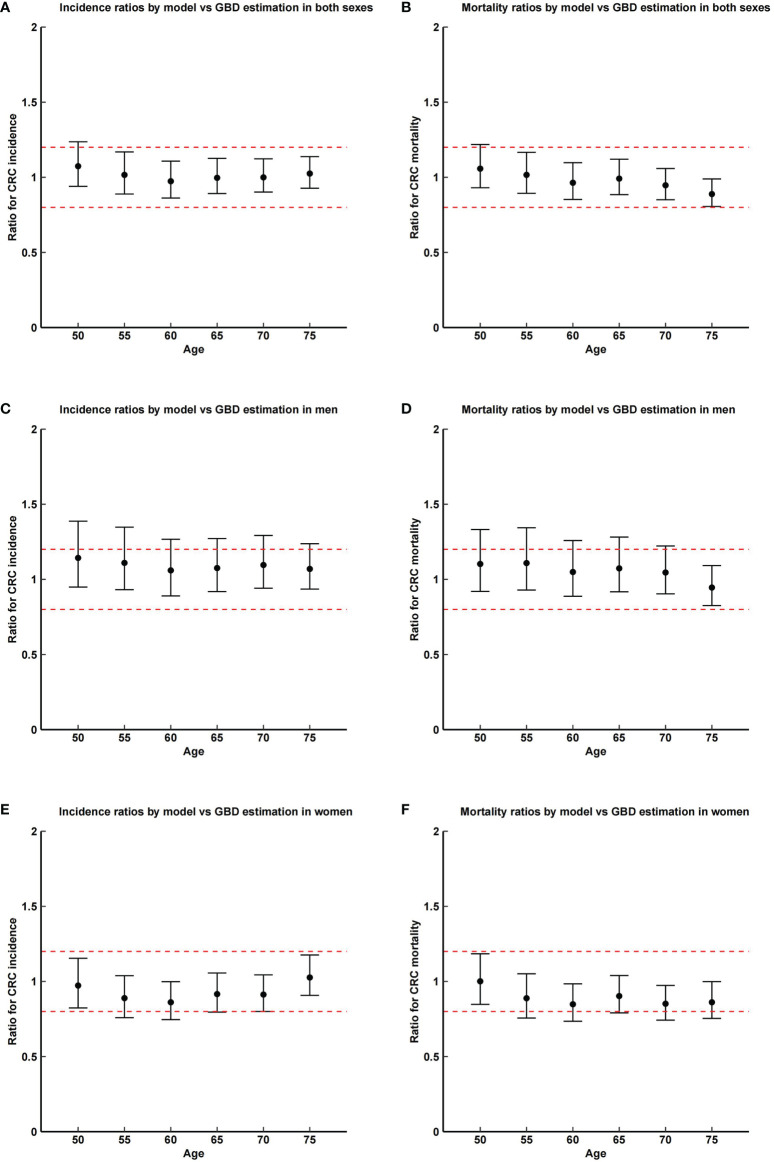
Ratios and corresponding CI of model-predicted incidence and mortality of colorectal cancer with the registry-based estimations in China in 2015. Dashed lines show the margin of testing for equivalence (0.2). GBD, global burden of disease. **(A)** incidence ratios by model vs. GBD estimation in both sexes; **(B)** mortality ratios by model vs. GBD estimation in both sexes; **(C)** incidence ratios by model vs. GBD estimation in men; **(D)** mortality ratios by model vs. GBD estimation in men; **(E)** incidence ratios by model vs. GBD estimation in women; **(F)** mortality ratios by model vs. GBD estimation in women.

**Figure 3** shows ratios of model-estimated incidence and mortality of CRC with the registry-based estimations in China (33). Model-predicted sex- and age-specific CRC incidence and mortality are mostly equivalent to the registry-based data. Estimates from the multilevel meta-analysis yielded RR of 1.01 (95% CIs, 0.92–1.10) and 0.94 (95% CIs, 0.81–1.08) for CRC incidence and mortality in both genders, RR of 1.08 (95% CIs, 1.01–1.16) and 1.02 (95% CIs, 0.91–1.13) for CRC incidence and mortality in men, and RR of 0.94 (95% CIs, 0.83–1.05) and 0.87 (95% CIs, 0.71–1.03) for CRC incidence and mortality in women. When tested for statistical equivalence at a 20% margin, except for mortality in women, other TOSTs yielded p-values <0.05 (see **Supplementary Figure 1**).

### Model Validation

Analogous to the screening effect seen in the UKFSS trial on sigmoidoscopy (see **Table 1**), the model-estimated cumulative incidence and mortality in the screening group were significantly lower in the control group. The HR of model-estimated incidence was 0.60, which was within the 95% CIs of observed HR for CRC incidence in the UKFSS trial (0.67, 95% CI, 0.60–0.76). The HR of CRC-related mortality was 0.51 in the model, which was comparable to the observation in the UKFSS trial (0.56, 95% CI, 0.45–0.69). Sigmoidoscopy screening had no significant effect on mortality due to non-CRC causes (see **Table 1**).

**Table 1:** Comparison of model-predicted outcome and the reported results in the UK Flexible Sigmoidoscopy Trial.

Metric		Incidence		All-cause mortality		Colorectal cancer mortality		Non-colorectal cancer causes mortality	
		UK	Model	UK	Model	UK	Model	UK	Model
Control group (n = 112,939)	Cases	1,818	1,333	13,768	17,467	515	617	13,131	16,850
	Person-years	1,218,334	1,206,179	1,224,523	1,210,276	1,224,523	1,210,276	1,224,523	1,210,276
	Rate (per 100,000 person-years; 95% CI)	149 (143–156)	111	1,124 (1,106–1,143)	1,443	52 (48–56)	51	1,072 (1,054–1,091)	1,392
Screening group (n = 40,621)	Cases	445	291	4,062	6,176	200	113	3,935	6,063
	Person-years	444,721	434,867	446,854	435,983	446,854	435,983	446,854	435,983
	Rate (per 100,000 person-years; 95% CI)	100 (91–110)	67	909 (881–937)	1,417	28 (24–34)	26	881 (854–909)	1,391
Hazard ratio (95% CI)		0.67 (0.60–0.76)	0.60	0.95 (0.91–1.00)	0.98	0.56 (0.45–0.69)	0.51	0.98 (0.93–1.03)	1.00

### Model Application

**Figure 4:**
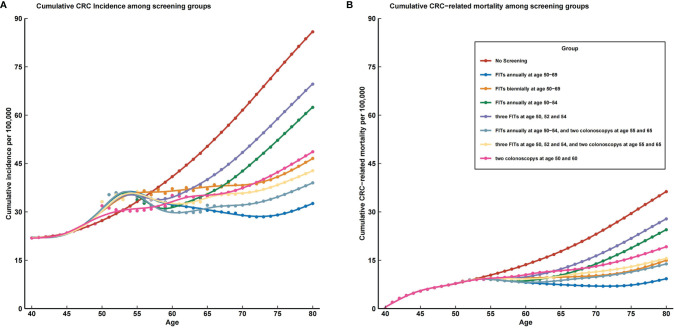
The prevalence trends of colorectal cancer incidence and mortality in all screening strategies compared with no screening. CRC, colorectal cancer; FIT, fecal immunochemical test. **(A)** cumulative CRC incidence among screening groups; **(B)** cumulative CRC related mortality among screening groups.

**Figure 4** shows the estimated results for the hypothetical screening scenarios. The detailed cumulative incidence and mortality of CRC of different ages in 8 scenarios are shown in **Table 2**. All screening strategies could reduce CRC incidence and mortality in China compared to no screening. Separately, the predicted HR of incident CRC at age 80 years was 0.380 for scenario 2 (FITs annually at age 50–69), 0.565 for scenario 3 (FITs biennially at age 50–69), 0.722 for scenario 4 (FITs annually at age 50–54), 0.825 for scenario 5 (three FITs at age 50, 52, and 54), 0.450 for scenario 6 (FITs annually at age 50–54, and two colonoscopies at age 55 and 65), 0.511 for scenario 7 (three FITs at age 50, 52, and 54, and two colonoscopies at age 55 and 65), and 0.555 for scenario 8 (two colonoscopies at age 50 and 60). The detailed information is shown in **Supplementary Figure 2** and **Supplementary Table 5**. Accordingly, the predicted HRs of CRC-related death at age 80 years for 7 screening scenarios were 0.242, 0.412, 0.685, 0.783, 0.384, 0.433, and 0.524 (see **Supplementary Figure 3** and **Supplementary Table 6**).

**Table 2:** Cumulative colorectal cancer incidence and mortality (/100,000) at different ages in 8 screening scenarios.

Scenarios		Age								
		40	45	50	55	60	65	70	75	80
Cumulative incidence of colorectal cancer	Scenario 1	13.01	20.61	25.09	32.50	41.56	51.00	60.94	73.96	86.16
	Scenario 2	13.01	20.61	31.15	34.48	31.83	30.09	28.93	29.42	32.74
	Scenario 3	13.01	20.61	31.15	34.79	38.41	38.02	39.78	42.88	48.68
	Scenario 4	13.01	20.61	31.15	33.07	31.74	35.48	43.03	52.55	62.17
	Scenario 5	13.01	20.61	31.15	34.79	35.17	40.34	48.28	58.67	71.09
	Scenario 6	13.01	20.61	31.15	34.09	30.21	32.63	33.24	35.49	38.79
	Scenario 7	13.01	20.61	31.15	36.71	33.25	36.67	35.88	38.28	44.06
	Scenario 8	13.01	20.61	30.42	29.82	35.91	35.25	37.01	41.46	47.85
Cumulative mortality of colorectal cancer	Scenario 1	0.00	4.86	7.35	10.12	13.57	18.67	24.63	31.31	38.65
	Scenario 2	0.00	4.86	7.35	8.72	7.79	7.02	6.70	7.25	9.35
	Scenario 3	0.00	4.86	7.35	9.55	9.80	10.31	10.69	12.47	15.91
	Scenario 4	0.00	4.86	7.35	8.72	8.67	11.72	14.71	20.42	26.47
	Scenario 5	0.00	4.86	7.35	9.55	10.09	13.36	17.83	23.88	30.26
	Scenario 6	0.00	4.86	7.35	8.72	8.23	9.35	10.24	12.48	14.83
	Scenario 7	0.00	4.86	7.35	9.55	9.60	11.07	12.47	13.96	16.75
	Scenario 8	0.00	4.86	7.35	9.10	10.58	12.16	14.10	16.75	20.25

Scenario 1, no screening; scenario 2, fecal immunochemical tests (FITs) annually at age 50–69; scenario 3, FITs biennially at age 50–69; scenario 4, FITs annually at age 50–54; scenario 5, three FITs at age 50, 52, and 54; scenario 6, FITs annually at age 50–54, and two colonoscopies at age 55 and 65; scenario 7, three FITs at age 50, 52, and 54, and two colonoscopies at age 55 and 65; scenario 8, two colonoscopies at age 50 and 60.

Based on the magnitude of reduction in incidence, scenario 2 (FITs annually at age 50–69) is the optimal screening strategy among all scenarios (see **Figure 4A**). This strategy would reduce the cumulative CRC incidence by 53% by age 70 and 62% by age 80. As the most commonly implemented CRC screening strategy, two colonoscopies at age 50 and 60 would reduce the cumulative CRC incidence by 46% by age 70 and 45% by age 80. Besides the optimal scenario, scenario 6 (FITs annually at 50–54 and two colonoscopies at age 55 and 65) and scenario 7 (three FITs at age 50, 52, and 54, and two colonoscopies at age 55 and 65) also prevent more CRC than does a one-time colonoscopy screening strategy.

The screening effects were similarly based on the magnitude of reduction in mortality except for the screening scenario 3 (FITs biennially at age 50–69), which could prevent more CRC-related death than one-time colonoscopy screening strategy (see **Figure 4B**). Moreover, screening scenario 3 also received a better effect than screening scenario 7 (three FITs at age 50, 52, and 54, and two colonoscopies at age 55 and 65). As the optimal strategy, scenario 2 (FITs annually at age 50–69) would reduce the cumulative CRC mortality by 73% by age 70 and 76% by age 80. Adopting two colonoscopies at age 50 and 60 would reduce the cumulative CRC mortality by 43% by age 70 and 48% by age 80.

### Sensitivity Analyses

Univariate sensitivity analyses revealed that the results remained largely stable over the plausible range of each parameter. The estimated HRs of CRC incidence at age 80 for different screening scenarios compared with no screening are shown in **Supplementary Table 5**. In most analyses, scenario 2 (FITs annually at age 50–69) was the optimal choice for CRC screening, followed by scenario 6 (FITs annually at age 50–54, and two colonoscopies at age 55 and 65). As shown in **Supplementary Table 6**, the HRs of CRC mortality at age 80 for different screening scenarios compared with no screening were nearly unchanged with scenario 2 being optimal.

## Discussion

Our study comprehensively described the newly developed MIMIC-CRC. First, we illustrated the construction, calibration, and assumption that demonstrated the feasibility and validity of the model. We found a large majority of agreement between the model-estimated and observed prevalence of colorectal lesions in a large population-based CRC screening program in China and highly consistent model- and registry-derived sex- and age-specific cumulative CRC incidence and mortality. Second, we examined the external validity of MIMIC-CRC, which showed a similar pattern in reduction of incidence and mortality of CRC for a hypothetical sigmoidoscopy screening scenario as compared to a realistic screening RCT (UKFSS trial). Lastly, we applied the validated model in the comparison of different CRC screening strategies, which revealed that scenario 2 (FITs annually at age 50–69) and scenario 6 (FITs annually at age 50–54, and two colonoscopies at age 55 and 65) obtained the most effectiveness of screening. Overall, our results indicated that MIMIC-CRC was a useful tool in simulating the natural history of CRC and evaluating the long-term effects of CRC screening.

This work, to our knowledge, is the first natural history-based, well-calibrated, and well-validated multistate microsimulation for CRC screening in China. The structure of MIMIC-CRC is according to the recognized adenoma-carcinoma pathway of CRC development, which has been proved to be viable in the previous model implementation in Germany (10). Unlike the models in CISNET (34), changes in polyp size are not used to present disease progression in MIMIC-CRC, because colorectal precancerous lesions are mostly recorded by classification including AA (adenoma ≥ 10 mm, or with high-grade dysplasia, or villous component) and NAA (adenoma, which does not meet the criteria of AA) in China. Apart from initial prevalence and death probabilities, which could be obtained from real-world studies, the accuracy of calibration for other transition parameters largely determines the applicability of the model. Calibration results in MIMIC-CRC, under the reliable model framework, illustrated the model’s ability to predict colorectal neoplasm prevalence and cumulative CRC incidence and mortality for up to 40 years. In particular, a large majority (overall, 124 out of 140) of model-predicted colorectal lesion prevalence were within the 95% CIs of observed prevalence in a large-scale population-based screening colonoscopy program in China in 2012–2015. In addition, in the multi-level meta-analysis of ratios of model-estimated incidence and mortality of CRC with the registry-based estimations, most tests for statistical equivalence at a 20% margin yielded p-values <0.05, except for mortality in women, which might indicate the underestimation of death probability in women.

After the calibration of parameters, additional evidence to support the validity of MIMIC-CRC comes from the simulation of a hypothetical sigmoidoscopy screening RCT, which yielded a comparable outcome to the UKFSS trial. In the control group, model estimation showed a pattern of lower CRC incidence, approximate CRC mortality, and higher all-cause mortality compared with the outcome in the UKFSS trial, which was consistent with the difference of CRC incidence and mortality between China and the United Kingdom in reality. From the perspective of the effect of sigmoidoscopy screening, the outcomes in MIMIC-CRC and UKFSS trials yielded a similar magnitude of reduction in all dimensions.

Based on the well-calibrated and validated model, we finally conducted a comparative analysis of different CRC screening scenarios in China to demonstrate the application of MIMIC-CRC. Our base-case results suggested that adopting FIT or colonoscopy for CRC screening would be more effective than no screening regardless of the screening strategy, and the result was stable in univariate sensitivity analyses. In the base case, scenario 2 (FITs annually at age 50–69) and scenario 6 (FITs annually at age 50–54, and two colonoscopies at age 55 and 65) are the two optimal choices for CRC screening, which were consistent with previous studies (35, 36). Zhong et al. (36) conducted a meta-analysis on efficacy and cost-effectiveness of FIT versus colonoscopy in colorectal cancer screening, which showed that annual or biennial FIT was very cost-effective compared with colonoscopy every 10 years. In Germany, people are offered 5 annual FITs at ages 50–54, followed by a first screening colonoscopy at age 55 if all of these FITs were negative (35), which is the same as scenario 6 in this study. The CRC screening strategy in Germany has been proved to be cost-effective, and our base case results also showed that this strategy ranks second among all screening choices, which even reached first in some cases in sensitivity analyses.

Although extensive efforts have been made to ensure the validity of the MIMIC-CRC model, it still has several limitations. First, due to the restriction by the availability of cancer statistics data in China, we obtained adenoma incidence and annual transition probabilities of states from other literature and calibrated the parameters by data from two sources, which have been proved to be feasible. Second, we assumed that all CRC arises from the adenoma-carcinoma pathway. In fact, 10%–20% of CRC cases progress by the serrated neoplasia pathway, and 2%–7% of CRC cases progress by the microsatellite instability (19). Greuter et al. (9) developed a model that explicitly includes serrated pathways based on the data from the Dutch COlonoscopy versus COlonography Screening (COCOS) trial, which appeared to have a relatively high incidence. Third, some currently available simulation models used validated risk prediction models to estimate individual cancer risk, which can reduce the misclassification (37). Our model cannot adopt this way for the time being due to the lack of a recognized prediction model in China. Fourth, the transition probability from adenoma to cancer does not vary by location within the colon and rectum, which have been proven to have different incidences and prognoses (19). Fifth, the birth cohort effect was neglected in the model due to the lack of CRC screening cohort in China. The present cohort can only represent the situation of the population born in 1975. Sixth, surveillance after colonoscopy was not included in the analyses. The surveillance is vital for the early diagnosis of adenoma recurrence after polypectomy, which can be further analyzed separately using the optimized model. Seventh, the cost and utility were not considered in the present analysis, because our model temporarily aimed to elucidate the efficacy of CRC screening strategies, which fully disclose the effect of CRC screening regardless of costs and medical resources. With the constant improvement of MIMIC-CRC, the model could be used to evaluate the cost-effectiveness of existing screening strategies (38), optimize the details of existing screening and surveillance modalities (39, 40), and explore the impact of social (41), economic (42), and disease intervention (43, 44) in CRC screening. While the model is not perfect currently, it can be extended in a variety of ways when data are supportive.

To sum up, we developed a multistate microsimulation model (MIMIC-CRC), which has been well-calibrated and well-validated, and we demonstrated good translational potential in solving issues regarding the comparative effectiveness of different screening strategies. The MIMIC-CRC model may therefore represent a valid tool to further decision-making in the CRC prevention area.

## Data Availability Statement

The data analyzed in this study is subject to the following licenses/restrictions: The dataset is private. Requests to access these datasets should be directed to BL, lb838744529@126.com.

## Author Contributions

MD and HC conceptualized and designed the study. HC, LW, and BL participated in the construction of the model. BL, LW, ML, YZ, JC, and CL participated in the acquisition and calibration of parameters. BL participated in the statistical analysis and drafted the manuscript. All authors critically revised the manuscript and approved the final manuscript.

## Funding

This work was supported by the National Natural Science Foundation of China (82173606), the Natural Science Foundation of Beijing Municipality (7202169), and the Beijing Nova Program of Science and Technology (Z191100001119065).

## Conflict of Interest

The authors declare that the research was conducted in the absence of any commercial or financial relationships that could be construed as a potential conflict of interest.

## Publisher’s Note

All claims expressed in this article are solely those of the authors and do not necessarily represent those of their affiliated organizations, or those of the publisher, the editors and the reviewers. Any product that may be evaluated in this article, or claim that may be made by its manufacturer, is not guaranteed or endorsed by the publisher.
