# Supplementary material for: Microsimulation Model for Prevention and Intervention of Coloretal Cancer in China (MIMIC-CRC): Development, Calibration, Validation, and Application
Source: Front Oncol. 2022 Apr 22;12:883401. doi: 10.3389/fonc.2022.883401 (PMC9072786; doi:10.3389/fonc.2022.883401)
Supplement: Supplementary file 1 [file DataSheet_1.docx]

Supplementary Material

# Supplementary Figures and Tables

## Supplementary Figures

## **
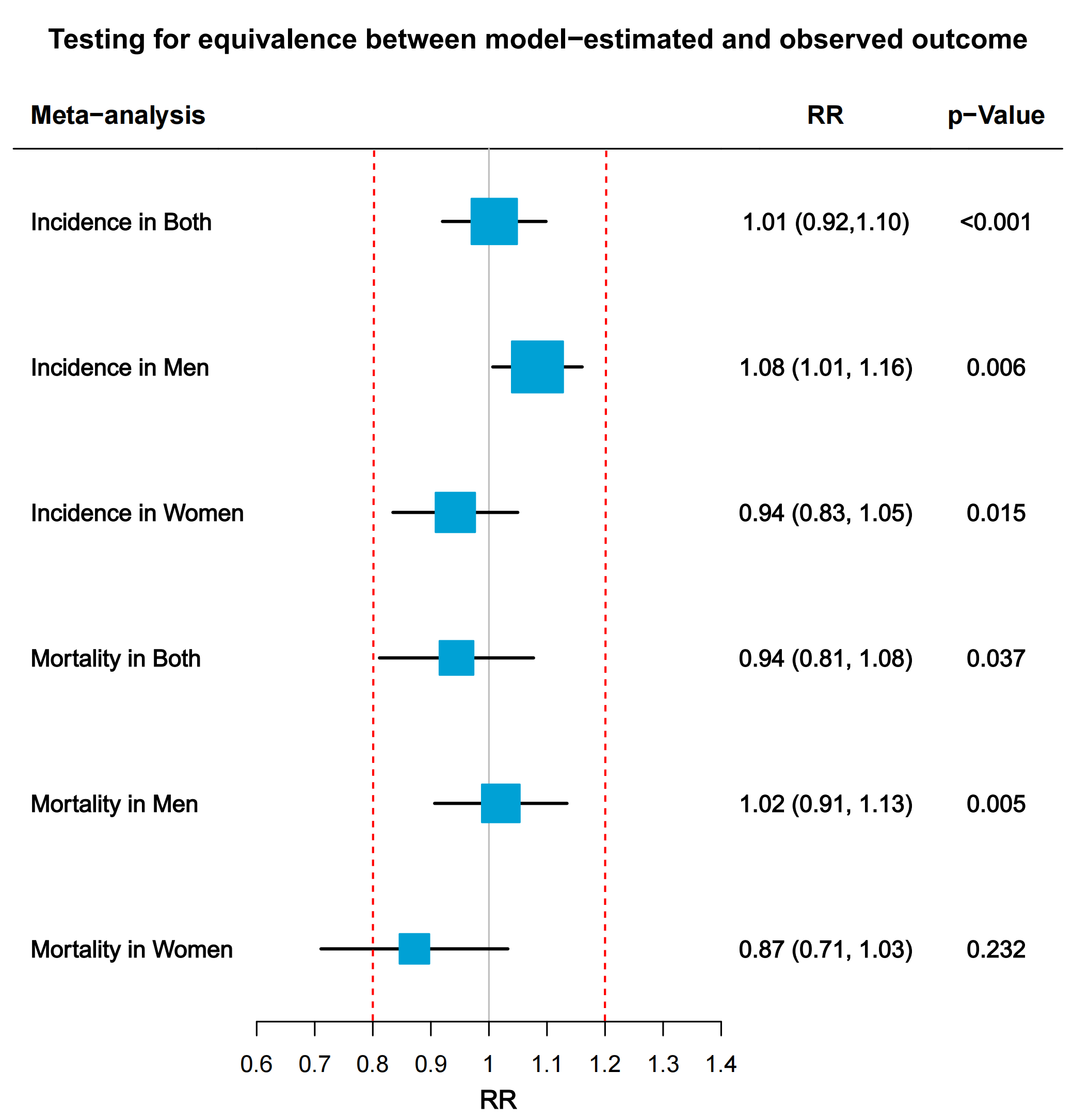
**

## **Supplementary Figure 1.** Testing for equivalence between model-estimated and observed outcomes by applying two one-sided t-tests (TOST). Shown are the meta-analyses estimates and 90% confidence intervals for the summarized rate ratios at ages 50-75 for colorectal cancer incidence and mortality. RR, rate ratio.

**
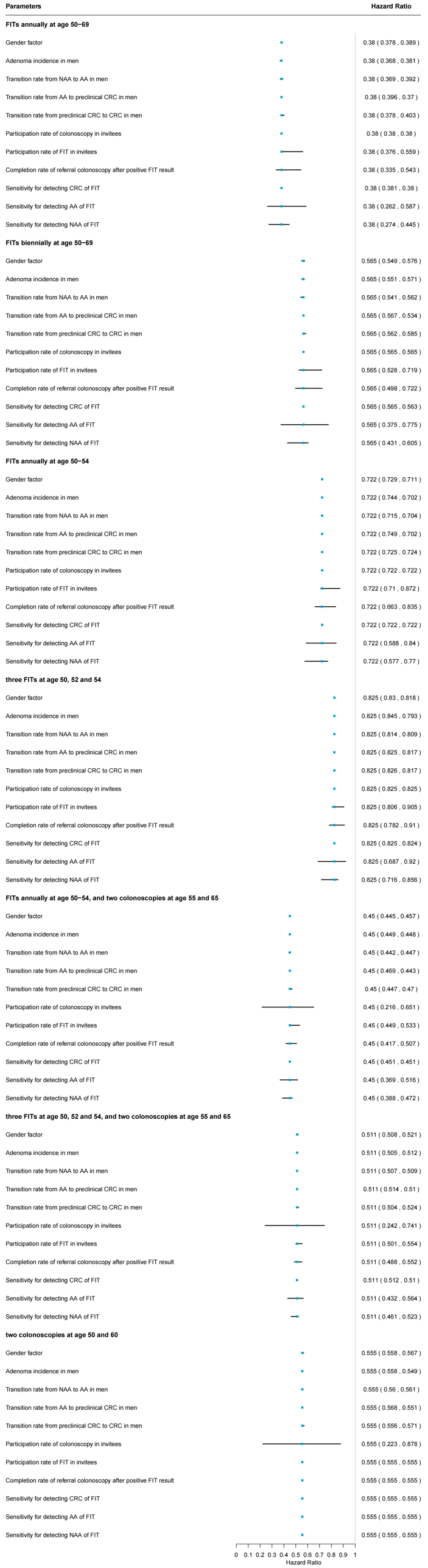
**

**
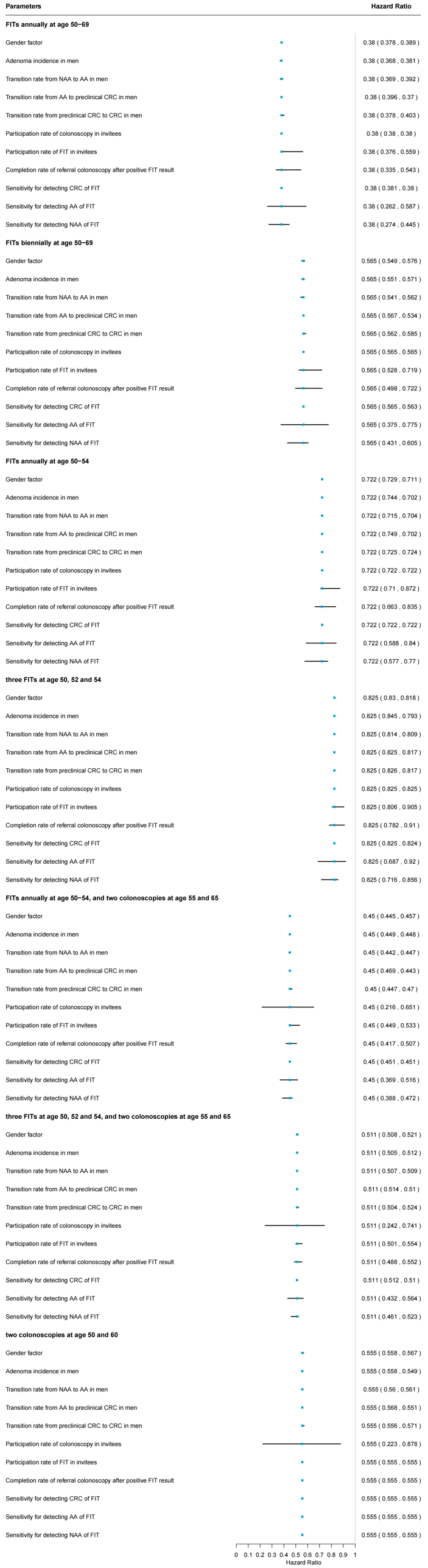
Supplementary Figure 2.** Forest plot for hazard ratio of colorectal cancer incidence at age 80 for different screening scenarios with no screening in univariate sensitivity analyses.

**
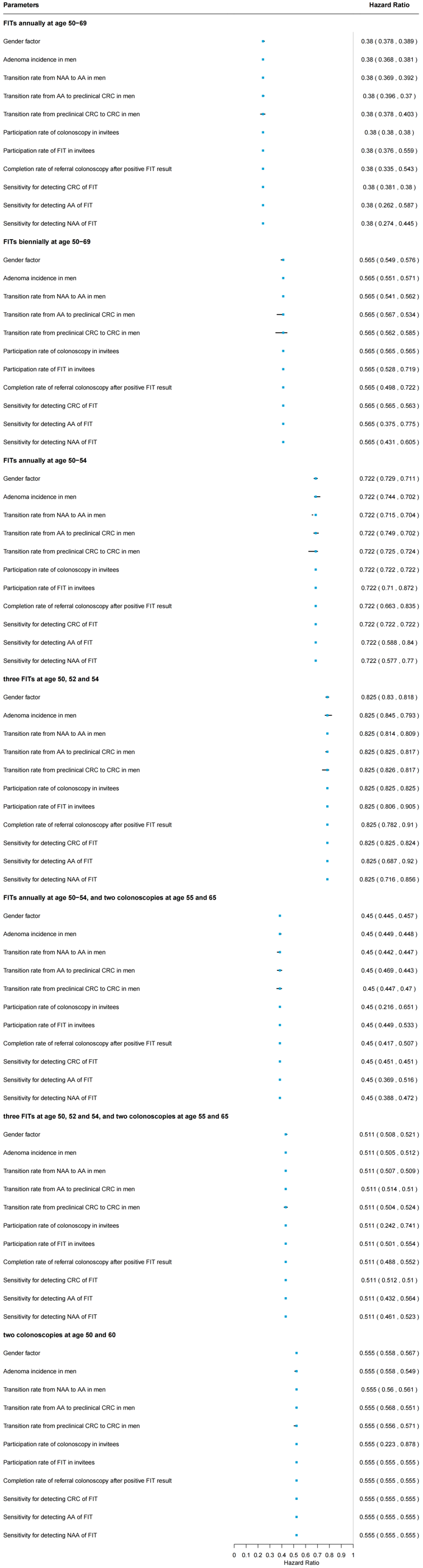
**

**
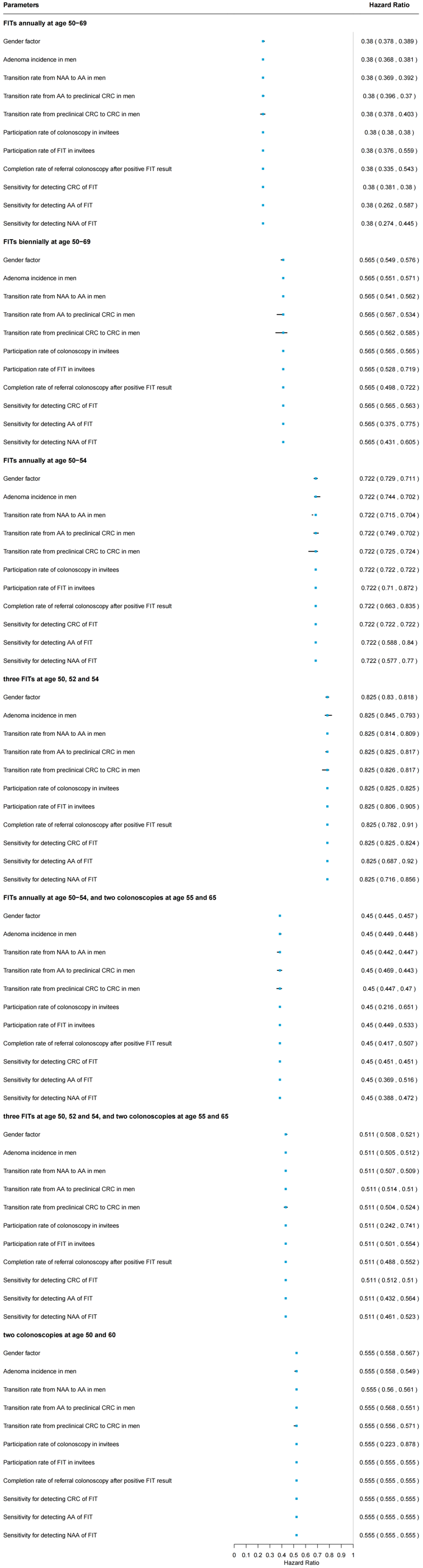
Supplementary Figure 3.** Forest plot for hazard ratio of colorectal cancer mortality at age 80 for different screening scenarios with no screening in univariate sensitivity analyses.

## Supplementary Tables

**Supplemental Table 1.** Estimates of natural history parameters used in the model

| Parameters | Base case value | Reference |
| --- | --- | --- |
| Initial prevalence of colorectal lesion in men |  | [1] |
| No colorectal lesion | 0.9208 |  |
| NAA | 0.0625 |  |
| AA | 0.0167 |  |
| CRC | 0.0000 |  |
| Initial prevalence of colorectal lesion in women |  |  |
| No colorectal lesion | 0.9623 |  |
| NAA | 0.0257 |  |
| AA | 0.0103 |  |
| CRC | 0.0017 |  |
| Adenoma incidence in men ^a^ | 0.008 | [2] |
| Transition probability from NAA to AA in men ^a^ | 0.035 |  |
| Transition probability from AA to preclinical CRC in men ^a^ | 0.020 |  |
| Transition probability from preclinical CRC to CRC in men ^a^ | 0.400 |  |
| Gender factor ^a, b^ | 0.800 |  |
| Mortality from screening-detected CRC in men ^c^ | | [3] |
| Year 1 after diagnosis | 0.050 |  |
| Year 2 after diagnosis | 0.024 |  |
| Year 3 after diagnosis | 0.023 |  |
| Year 4 after diagnosis | 0.016 |  |
| Year 5 after diagnosis | 0.013 |  |
| Mortality from screening-detected CRC in women ^c^ | |  |
| Year 1 after diagnosis | 0.040 |  |
| Year 2 after diagnosis | 0.021 |  |
| Year 3 after diagnosis | 0.014 |  |
| Year 4 after diagnosis | 0.010 |  |
| Year 5 after diagnosis | 0.007 |  |
| Mortality from symptom-detected CRC in men ^c^ | |  |
| Year 1 after diagnosis | 0.215 |  |
| Year 2 after diagnosis | 0.101 |  |
| Year 3 after diagnosis | 0.096 |  |
| Year 4 after diagnosis | 0.069 |  |
| Year 5 after diagnosis | 0.055 |  |
| Mortality from symptom-detected CRC in women ^c^ | |  |
| Year 1 after diagnosis | 0.225 |  |
| Year 2 after diagnosis | 0.117 |  |
| Year 3 after diagnosis | 0.081 |  |
| Year 4 after diagnosis | 0.052 |  |
| Year 5 after diagnosis | 0.036 |  |
| Age factor ^a^ |  |  |
| Age 40 to 44 | 0.800 | Calibration Obtained |
| Age 45 to 49 | 0.850 |  |
| Age 50 to 54 | 0.900 |  |
| Age 55 to 59 | 0.950 |  |
| Age 60 to 64 | 1.000 |  |
| Age 65 to 69 | 1.100 |  |
| Age 70 to 74 | 1.200 |  |
| Age 75 to 79 | 1.300 |  |
| Background mortality in men | | [4] |
| Age 40 to 44 | 0.002244880 |  |
| Age 45 to 49 | 0.003081100 |  |
| Age 50 to 54 | 0.006161740 |  |
| Age 55 to 59 | 0.006937325 |  |
| Age 60 to 64 | 0.012952485 |  |
| Age 65 to 69 | 0.022345751 |  |
| Age 70 to 74 | 0.033852437 |  |
| Age 75 to 79 | 0.053083914 |  |
| Age 80 to 84 | 0.089359426 |  |
| Background mortality in women | |  |
| Age 40 to 44 | 0.000888705 |  |
| Age 45 to 49 | 0.001275686 |  |
| Age 50 to 54 | 0.002868378 |  |
| Age 55 to 59 | 0.003080047 |  |
| Age 60 to 64 | 0.006534854 |  |
| Age 65 to 69 | 0.012499425 |  |
| Age 70 to 74 | 0.021169610 |  |
| Age 75 to 79 | 0.035738350 |  |
| Age 80 to 84 | 0.066797537 |  |

^a^ Parameters which were calibrated based on the data from literature.

^b^ Gender factor is the reduction index for parameters in women compared to men.

^c^ Parameters are mortality in Germany multiplied by 1.1 to reach the consistency with the observed 5-year relative survival rate of CRC in China (56.8% after adjusted vs. 56.9% in reality).

Abbreviations: AA: advanced adenoma; CRC: colorectal cancer; NAA: non-advanced adenoma.

**Supplemental Table 2.** The age- and sex-specified mortality (/100,000) of all-cause, colorectal cancer and non-colorectal-cancer in China in 2015

| Age | Men | | |  | Women | | |  | Both | | |
| --- | --- | --- | --- | --- | --- | --- | --- | --- | --- | --- | --- |
|  | All death | CRC | Other Death |  | All death | CRC | Other Death |  | All death | CRC | Other Death |
| 40-44 | 227.64 | 2.90 | 224.74 |  | 91.43 | 2.52 | 88.91 |  | 160.19 | 2.71 | 157.48 |
| 45-49 | 313.63 | 5.01 | 308.62 |  | 131.01 | 3.36 | 127.65 |  | 222.11 | 4.18 | 217.93 |
| 50-54 | 629.37 | 11.29 | 618.08 |  | 294.20 | 6.95 | 287.25 |  | 465.64 | 9.17 | 456.47 |
| 55-59 | 709.95 | 13.80 | 696.15 |  | 316.92 | 8.44 | 308.48 |  | 514.09 | 11.13 | 502.96 |
| 60-64 | 1331.68 | 27.97 | 1303.71 |  | 671.62 | 15.99 | 655.63 |  | 1005.25 | 22.04 | 983.21 |
| 65-69 | 2306.99 | 47.07 | 2259.92 |  | 1285.12 | 27.30 | 1257.82 |  | 1798.06 | 37.22 | 1760.84 |
| 70-74 | 3509.70 | 65.83 | 3443.87 |  | 2179.07 | 39.38 | 2139.69 |  | 2841.84 | 52.56 | 2789.28 |
| 75-79 | 5547.44 | 92.96 | 5454.48 |  | 3699.34 | 60.08 | 3639.26 |  | 4578.80 | 75.73 | 4503.07 |
| 80-84 | 9493.60 | 132.90 | 9360.70 |  | 7004.32 | 91.01 | 6913.31 |  | 8118.88 | 109.76 | 8009.12 |
| 85~ | 19637.55 | 195.48 | 19442.07 |  | 16574.66 | 121.68 | 16452.98 |  | 17751.38 | 150.03 | 17601.35 |

Abbreviations: CRC: colorectal cancer.

**Supplemental Table 3.** Estimates of screening parameters used in the model

| Parameters | Base case value | Reference |
| --- | --- | --- |
| Participation rate of colonoscopy in invitees | 0.425 | [5] |
| Participation rate of FIT in invitees | 0.94 |  |
| Completion rate of referral colonoscopy after positive sigmoidoscopy result | 0.96 | [6] |
| Completion rate of referral colonoscopy after positive FIT result | 0.76 | [5] |
| Sensitivity for detecting CRC for sigmoidoscopy | 0.61 | [7] |
| Sensitivity for detecting adenoma for sigmoidoscopy | 0.59 |  |
| Specificity for sigmoidoscopy | 0.92 |  |
| Sensitivity for detecting CRC for FIT | 0.76 | [8] |
| Sensitivity for detecting advanced adenoma for FIT | 0.26 |  |
| Sensitivity for detecting non-advanced adenoma for FIT | 0.05 | [9] |
| Specificity for FIT | 0.95 | [8] |

Abbreviations: CRC: colorectal cancer; FIT, fecal immunochemical test.

**Supplemental Table 4.** The range of parameters in univariate sensitivity analyses

| Parameters | Base case value | Range |
| --- | --- | --- |
| Gender factor | 0.800 | 0.400-1.000 |
| Adenoma incidence in men | 0.008 | 0.004-0.016 |
| Transition rate from NAA to AA in men | 0.035 | 0.020-0.050 |
| Transition rate from AA to preclinical CRC in men | 0.020 | 0.010-0.030 |
| Transition rate from preclinical CRC to CRC in men | 0.400 | 0.200-0.600 |
| Participation rate of colonoscopy in invitees | 0.425 | 0.100-1.000 |
| Participation rate of FIT in invitees | 0.940 | 0.500-1.000 |
| Completion rate of referral colonoscopy after positive FIT result | 0.760 | 0.400-1.000 |
| Sensitivity for detecting CRC of FIT | 0.760 | 0.500-1.000 |
| Sensitivity for detecting AA of FIT | 0.260 | 0.100-0.600 |
| Sensitivity for detecting NAA of FIT | 0.050 | 0.000-0.300 |

Abbreviations: AA: advanced adenoma; CRC: colorectal cancer; FIT, fecal immunochemical test; NAA: non-advanced adenoma.

**Supplemental Table 5.** Hazard ratio of colorectal cancer incidence at age 80 for different screening scenarios with no screening in univariate sensitivity analyses

| Parameters | | No Screening | FITs annually at age 50-69 | FITs biennially at age 50-69 | FITs annually at age 50-54 | three FITs at age 50, 52 and 54 | FITs annually at age 50-54, and two colonoscopies at age 55 and 65 | three FITs at age 50, 52 and 54, and two colonoscopies at age 55 and 65 | two colonoscopies at age 50 and 60 |
| --- | --- | --- | --- | --- | --- | --- | --- | --- | --- |
| Base case | | 1.000 | 0.380# | 0.565 | 0.722 | 0.825 | 0.450* | 0.511 | 0.555 |
| Gender factor | lower limit | 1.000 | 0.389# | 0.576 | 0.711 | 0.818 | 0.457* | 0.521 | 0.567 |
|  | upper limit | 1.000 | 0.378# | 0.549 | 0.729 | 0.830 | 0.445* | 0.508 | 0.558 |
| Adenoma incidence in men | lower limit | 1.000 | 0.381# | 0.571 | 0.702 | 0.793 | 0.448* | 0.512 | 0.549 |
|  | upper limit | 1.000 | 0.368# | 0.551 | 0.744 | 0.845 | 0.449* | 0.505 | 0.558 |
| Transition rate from NAA to AA in men | lower limit | 1.000 | 0.392# | 0.562 | 0.704 | 0.809 | 0.447* | 0.509 | 0.561 |
|  | upper limit | 1.000 | 0.369# | 0.541 | 0.715 | 0.814 | 0.442* | 0.507 | 0.560 |
| Transition rate from AA to preclinical CRC in men | lower limit | 1.000 | 0.370# | 0.534 | 0.702 | 0.817 | 0.443* | 0.510 | 0.551 |
|  | upper limit | 1.000 | 0.396# | 0.567 | 0.749 | 0.825 | 0.469* | 0.514 | 0.568 |
| Transition rate from preclinical CRC to CRC in men | lower limit | 1.000 | 0.403# | 0.585 | 0.724 | 0.817 | 0.470* | 0.524 | 0.571 |
|  | upper limit | 1.000 | 0.378# | 0.562 | 0.725 | 0.826 | 0.447* | 0.504 | 0.556 |
| Participation rate of colonoscopy in invitees | lower limit | 1.000 | 0.380# | 0.565* | 0.722 | 0.825 | 0.651 | 0.741 | 0.878 |
|  | upper limit | 1.000 | 0.380 | 0.565 | 0.722 | 0.825 | 0.216# | 0.242* | 0.223 |
| Participation rate of FIT in invitees | lower limit | 1.000 | 0.559 | 0.719 | 0.872 | 0.905 | 0.533# | 0.554* | 0.555 |
|  | upper limit | 1.000 | 0.376# | 0.528 | 0.710 | 0.806 | 0.449* | 0.501 | 0.555 |
| Completion rate of referral colonoscopy after positive FIT result | lower limit | 1.000 | 0.543* | 0.722 | 0.835 | 0.910 | 0.507# | 0.552 | 0.555 |
|  | upper limit | 1.000 | 0.335# | 0.498 | 0.663 | 0.782 | 0.417* | 0.488 | 0.555 |
| Sensitivity for detecting CRC of FIT | lower limit | 1.000 | 0.380# | 0.563 | 0.722 | 0.824 | 0.451* | 0.510 | 0.555 |
|  | upper limit | 1.000 | 0.381# | 0.565 | 0.722 | 0.825 | 0.451* | 0.512 | 0.555 |
| Sensitivity for detecting AA of FIT | lower limit | 1.000 | 0.587 | 0.775 | 0.840 | 0.920 | 0.516# | 0.564* | 0.555 |
|  | upper limit | 1.000 | 0.262# | 0.375 | 0.588 | 0.687 | 0.369* | 0.432 | 0.555 |
| Sensitivity for detecting NAA of FIT | lower limit | 1.000 | 0.445# | 0.605 | 0.770 | 0.856 | 0.472* | 0.523 | 0.555 |
|  | upper limit | 1.000 | 0.274# | 0.431 | 0.577 | 0.716 | 0.388* | 0.461 | 0.555 |

Abbreviations: AA: advanced adenoma; CRC: colorectal cancer; FIT, fecal immunochemical test; NAA: non-advanced adenoma.

# The hazard ratio is the lowest among different screening scenarios.

* The hazard ratio is the second-lowest among different screening scenarios.

**Supplemental Table 6.** Hazard ratio of colorectal cancer mortality at age 80 for different screening scenarios with no screening in univariate sensitivity analyses

| Parameters | | No Screening | FITs annually at age 50-69 | FITs biennially at age 50-69 | FITs annually at age 50-54 | three FITs at age 50, 52 and 54 | FITs annually at age 50-54, and two colonoscopies at age 55 and 65 | three FITs at age 50, 52 and 54, and two colonoscopies at age 55 and 65 | two colonoscopies at age 50 and 60 |
| --- | --- | --- | --- | --- | --- | --- | --- | --- | --- |
| Base case | | 1.000 | 0.242# | 0.412 | 0.685 | 0.783 | 0.384* | 0.433 | 0.524 |
| Gender factor | lower limit | 1.000 | 0.246# | 0.394 | 0.671 | 0.770 | 0.386* | 0.440 | 0.524 |
|  | upper limit | 1.000 | 0.254# | 0.407 | 0.699 | 0.795 | 0.386* | 0.444 | 0.518 |
| Adenoma incidence in men | lower limit | 1.000 | 0.241# | 0.420 | 0.674 | 0.764 | 0.378* | 0.445 | 0.509 |
|  | upper limit | 1.000 | 0.237# | 0.410 | 0.721 | 0.819 | 0.394* | 0.439 | 0.530 |
| Transition rate from NAA to AA in men | lower limit | 1.000 | 0.254# | 0.409 | 0.655 | 0.775 | 0.362* | 0.450 | 0.525 |
|  | upper limit | 1.000 | 0.235# | 0.390 | 0.661 | 0.773 | 0.374* | 0.435 | 0.527 |
| Transition rate from AA to preclinical CRC in men | lower limit | 1.000 | 0.239# | 0.360* | 0.669 | 0.769 | 0.362 | 0.435 | 0.517 |
|  | upper limit | 1.000 | 0.251# | 0.417 | 0.708 | 0.776 | 0.400* | 0.437 | 0.530 |
| Transition rate from preclinical CRC to CRC in men | lower limit | 1.000 | 0.221# | 0.349* | 0.626 | 0.741 | 0.358 | 0.420 | 0.503 |
|  | upper limit | 1.000 | 0.259# | 0.444 | 0.701 | 0.795 | 0.399* | 0.447 | 0.519 |
| Participation rate of colonoscopy in invitees | lower limit | 1.000 | 0.242# | 0.412* | 0.685 | 0.783 | 0.603 | 0.691 | 0.879 |
|  | upper limit | 1.000 | 0.242 | 0.412 | 0.685 | 0.783 | 0.134# | 0.159* | 0.162 |
| Participation rate of FIT in invitees | lower limit | 1.000 | 0.432# | 0.618 | 0.848 | 0.908 | 0.465* | 0.510 | 0.524 |
|  | upper limit | 1.000 | 0.233# | 0.374 | 0.644 | 0.779 | 0.373* | 0.431 | 0.524 |
| Completion rate of referral colonoscopy after positive FIT result | lower limit | 1.000 | 0.412# | 0.612 | 0.831 | 0.895 | 0.455* | 0.495 | 0.524 |
|  | upper limit | 1.000 | 0.206# | 0.328* | 0.615 | 0.721 | 0.343 | 0.398 | 0.524 |
| Sensitivity for detecting CRC of FIT | lower limit | 1.000 | 0.273# | 0.446 | 0.701 | 0.790 | 0.400* | 0.437 | 0.524 |
|  | upper limit | 1.000 | 0.228# | 0.389 | 0.680 | 0.771 | 0.380* | 0.424 | 0.524 |
| Sensitivity for detecting AA of FIT | lower limit | 1.000 | 0.381# | 0.578 | 0.808 | 0.864 | 0.448* | 0.467 | 0.524 |
|  | upper limit | 1.000 | 0.180# | 0.279* | 0.555 | 0.646 | 0.315 | 0.365 | 0.524 |
| Sensitivity for detecting NAA of FIT | lower limit | 1.000 | 0.304# | 0.435 | 0.741 | 0.817 | 0.403* | 0.449 | 0.524 |
|  | upper limit | 1.000 | 0.164# | 0.306* | 0.517 | 0.672 | 0.309 | 0.379 | 0.524 |

Abbreviations: AA: advanced adenoma; CRC: colorectal cancer; FIT, fecal immunochemical test; NAA: non-advanced adenoma.

# The hazard ratio is the lowest among different screening scenarios.

* The hazard ratio is the second-lowest among different screening scenarios.

**References:**

1. Chen H, Li N, Ren J, Feng X, Lyu Z, Wei L, et al. Participation and yield of a population-based colorectal cancer screening programme in China. Gut. 2019;68(8):1450-1457.
2. Greuter MJ, Xu XM, Lew JB, Dekker E, Kuipers EJ, Canfell K, et al. Modeling the Adenoma and Serrated pathway to Colorectal CAncer (ASCCA). Risk Anal. 2014;34(5):889-910.
3. Heisser T, Weigl K, Hoffmeister M, Brenner H. Age-specific sequence of colorectal cancer screening options in Germany: a model-based critical evaluation. PLoS medicine. 2020;17(7): e1003194.
4. National Health and Family Planning Commission. China Health & Family Planning Statistics Yearbook 2016 (in Chinese). Beijing: Peking Union Medical College Press; 2016. p. 284-309.
5. Chen H, Lu M, Liu C, Zou S, Du L, Liao X, et al. Comparative evaluation of participation and diagnostic yield of colonoscopy vs fecal immunochemical test vs risk-adapted screening in colorectal cancer screening: interim analysis of a multicenter randomized controlled trial (TARGET-C). Am J Gastroenterol. 2020;115(8):1264-1274.
6. Atkin WS, Cook CF, Cuzick J, Edwards R, Northover JM, Wardle J, et al. Single flexible sigmoidoscopy screening to prevent colorectal cancer: baseline findings of a UK multicentre randomised trial. Lancet. 2002;359(9314):1291-1300.
7. Niedermaier T, Weigl K, Hoffmeister M, Brenner H. Diagnostic performance of flexible sigmoidoscopy combined with fecal immunochemical test in colorectal cancer screening: meta-analysis and modeling. Eur J Epidemiol. 2017;32(6):481-493.
8. Selby K, Levine EH, Doan C, Gies A, Brenner H, Quesenberry C, et al. Effect of sex, age, and positivity threshold on fecal immunochemical test accuracy: a systematic review and Meta-analysis. Gastroenterology. 2019;157(6):1494-1505.
9. Lu M, Zhang YH, Lu B, Cai J, Liu CC, Chen HD, et al. Head-to-head comparison of the test performance of self-administered qualitative vs. laboratory-based quantitative fecal immunochemical tests in detecting colorectal neoplasm. Chin Med J (Engl). 2021;134(11):1335-1344.
